# Gene Expression of Caenorhabditis elegans Neurons Carries Information on Their Synaptic Connectivity

**DOI:** 10.1371/journal.pcbi.0020167

**Published:** 2006-12-08

**Authors:** Alon Kaufman, Gideon Dror, Isaac Meilijson, Eytan Ruppin

**Affiliations:** 1 Interdisciplinary Center for Neural Computation, Hebrew University, Jerusalem, Israel; 2 School of Computer Science, The Academic College of Tel Aviv–Yaffo, Tel Aviv, Israel; 3 School of Mathematical Sciences, Tel Aviv University, Tel Aviv, Israel; 4 School of Computer Science and School of Medicine, Tel Aviv University, Tel Aviv, Israel; University College London, United Kingdom

## Abstract

The claim that genetic properties of neurons significantly influence their synaptic network structure is a common notion in neuroscience. The nematode Caenorhabditis elegans provides an exciting opportunity to approach this question in a large-scale quantitative manner. Its synaptic connectivity network has been identified, and, combined with cellular studies, we currently have characteristic connectivity and gene expression signatures for most of its neurons*.* By using two complementary analysis assays we show that the expression signature of a neuron carries significant information about its synaptic connectivity signature, and identify a list of putative genes predicting neural connectivity. The current study rigorously quantifies the relation between gene expression and synaptic connectivity signatures in the C. elegans nervous system and identifies subsets of neurons where this relation is highly marked. The results presented and the genes identified provide a promising starting point for further, more detailed computational and experimental investigations.

## Synopsis

The study of the genetic basis of the formation of neural connections in the nervous system (synaptogenesis) has been at the forefront of recent investigations in neuroscience. With the advancement of molecular biology research, many small-scale studies have identified specific genes and mechanisms involved in axon guidance and synaptogenesis. The nematode C. elegans provides an exciting opportunity to approach these issues in a computational large-scale manner. Its synaptic connectivity network has been identified, and, combined with information from gene expression studies, we now have neuronal connectivity and gene expression signatures for most of its neurons. Analyzing this data, Kaufman and colleagues show that the expression signature of a neuron carries significant information about its synaptic connectivity and can predict its neural targets in a statistically significant manner. The current study is the first, to our knowledge, to rigorously quantify and measure this relation. It identifies a putative list of genes that specify the neurons' connections which nicely conforms with the existing literature and leads to interesting new predictions.

## Introduction

It is an accepted common notion that genes play a major role in the formation of the nervous system; they specify neuronal cell types, help destine neurons into defined neural circuits, and provide important cues determining their communication [1,2]. Many studies have identified specific genes in the nematode C. elegans that disrupt the development of neural circuits. These genes are typically responsible for neuronal morphology, axon development, and synaptogenesis. Such findings include, e.g., axon guidance genes (*sax-3, unc-34,* and the netrin receptor *unc-40*) [3], attractive and repulsive interactions *(unc-6, unc-40,* and *unc-5)* [4–6], presynaptic input modulation *(unc-4, unc-37)* [7], presynaptic differentiation *(sad-1)* [8], and synaptic specificity genes *(syg-1, syg-2)* [9]. These findings have been based on specifically targeted studies, each designed to address a specific pathway, neuron type, receptor or transmitter (for reviews see [10–14]). Yet, it has been difficult to identify on a large scale mutations that determine the specific identity of synaptic connections (that is, to which other neurons each neuron is connected), mainly because synaptic specification is one of the last steps in a complex process of neuronal differentiation and axonal migration [11]. Sieburth et al. [15] presented the first large-scale screening for genes involved in the C. elegans neuromuscular junction. The study identified more than 100 novel genes that have specific functions in the transmission of signals across this junction. While the latter study was not aimed at identifying synaptic connectivity genes, it demonstrated the plausibility of addressing such questions in a large-scale manner. In a recent and related study, Varadan et al. [16] have applied an entropy minimization approach to the *C. elegans'* data to identify sets of synergistically interacting genes whose joint expression is common to most synapses and predicts neural connectivity, leaving aside the specific identity of the pre- and post-neurons. Our study differs from theirs both in its goals (identifying the genes which predict the specific whole connectivity pattern of a particular given neuron), and in its methods. It leads to the first quantitative characterization of the relation between the genetic properties of neurons and their synaptic connectivity, concomitantly addressing the majority of C. elegans neurons at large.

The existing C. elegans neural wiring diagram provides a “connectivity signature” for each neuron, specifying to which other neurons it is connected (R. M. Durbin (http://elegans.swmed.edu/parts/neurodata.txt), based on the classic work of White et al. [17] and Hall et al. [18]). Each neuron has also an “expression signature” extracted from WormBase (http://wormbase.org), specifying the genes directly associated with it (see Materials and Methods). Combined together, this data enables the investigation of the relation between expression and connectivity signatures across most of the C. elegans neurons. We specifically address two attributes of this relation: the first asks whether it is possible to predict a neuron's connectivity signature based solely on its expression signature. The second question is, to what extent do neurons with similar expression signatures have similar connectivity signatures? We show that the expression signatures of neurons carry significant information about their connectivity signatures, and further identify specific genes that play a major role in determining this relation. The gene sets we identify do not necessarily have a direct causal influence on synaptic connectivity and specificity; however, they provide putative gene targets for further experimental investigation.

Finally, we suggest a methodological way to study the relations between neurons' expression and connectivity signatures and their actual functional contribution to behavior. Quantifying these relations allows addressing a classical question in neuroscience; what dominates the functionality of a neural circuit—the local, genetic basis of the individual neurons, or the overall network structure determined by their connectivity?

## Results

For a majority of the C. elegans neurons, we obtained two types of data signatures (Materials and Methods): 1) the gene expression signature, describing which genes have expression patterns that are directly associated with a neuron, according to WormBase; and 2) the connectivity signature, describing the outgoing and incoming synaptic connections of each neuron to all other neurons in the network (focusing only on synaptic connections in which the direction is well-defined). To avoid a bias caused by the symmetric structure of the data (many of the neurons are situated bilaterally along the nematode body and head), we focused on the right side of the nematode (and including also neurons without a symmetrical companion), retaining 98 such neurons with both an expression and a connectivity signature (Table S1).

The natural starting point for investigating the relation between these two types of signatures revolves around two basic attributes: First, the prediction ability—that is, can the connectivity signatures be predicted based on the expression signatures? Second, a covariation correlation assay—which essentially measures to what extent are the neighborhood relations between neurons in one space (e.g., expression) similar to their neighborhood relations in the other space (e.g., synaptic connectivity). To study the first prediction question, we use a standard weighted K–nearest neighbor (KNN) prediction algorithm with multiclass targets (see Materials and Methods). Based on the expression signature of each neuron, this algorithm predicts its connectivity signature. The resulting prediction accuracy is measured in a conventional manner by the average area under the receiver operating characteristic (ROC) curve (AUC). The performance obtained was 0.594 and 0.601 in predicting the incoming and outgoing connectivity signatures, respectively (*p-*value = 10^−85^, and *p-*value = 10^−75^, respectively, with respect to the performance on randomly shuffled data). The predictor's AUC was moderate, probably reflecting the crude data in hand, but, nevertheless, it manifests a markedly statistically significant signal. To study the second question, we applied a covariation correlation assay (see Materials and Methods) to the 98 neurons, finding a Pearson correlation of 0.075 (*p-*value < 0.0001) between the gene expression neighborhood relations and the incoming connectivity neighborhood relations, and 0.176 (*p-*value < 0.0001) between the expression neighborhood relations and the outgoing synaptic neighborhood relations (the similarity measure we use for computing the pairwise similarity between neurons in each space is the 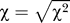 index, and Protocol S1 shows the correlations between the signatures when using alternative similarity measures). These low-magnitude but strongly significant correlations indicate that the neighborhood relations between neurons in the one space bear a moderate similarity to the neighborhood relations in the other space.

Importantly, these prediction and correlation levels are an average over all neurons. Indeed, examining individual neurons we do see significantly higher levels. We identified 15 presynaptic and 15 postsynaptic neurons for which the connectivity prediction accuracy manifest increased AUC levels. These levels were between 0.6 and 0.98, with high statistical significance after correcting for multiple hypotheses testing (Protocol S2 discusses this analysis in detail). Using a semiparametric statistical model allows one to reliably discriminate between AUC values obtained from the predictor-based distribution and the random distribution. In particular, this is of importance for the relatively rare cases achieving very high accuracy levels (see Protocol S3 for a detailed description of the model and its analysis). Based on the model, once a predictor's AUC is given, one can compute the ratio between the probabilities to achieve such an AUC from the predictor-based distribution and from a random predictor. For example, for neurons manifesting AUC values of 0.9 the ratio is 5.05 in the incoming connectivity case and 5.59 in the outgoing case. Similarly, in the covariation analysis we show that within subsets of neurons, divided according to neuron type (Table S1), some subsets show correlation levels higher than the correlation reported in the general case which remain statistically significant after correcting for multiple hypotheses testing. For example, the motor neurons manifest a correlation as high as 0.626 (data presented in Table 2 in Protocol S4).

Neuron cell type probably plays a major role in determining the connectivity properties of the cell, as distinct cell type–specific properties are determined by the combinatorial functions of multiple transcription factors [19–21]. Indeed, applying the prediction assay to predict the neuron type based on the expression signatures (where neurons are classified into a number of neuron types according to WormBase (Materials and Methods and Table S1)) shows a significant prediction capability (AUC = 0.923, *p-*value = 10^−20^)*.* Consequently, the relation between expression and connectivity signatures was further examined by the prediction and covariation correlation assays while controlling for specific information about cell types. The relations between the two signatures remained marked and significant, both for the incoming and outgoing signatures, with AUC = 0.599 (*p-*value = 10^−67^) and AUC = 0.611 (*p-*value = 10^−59^) in the prediction assays and correlations of 0.089 (*p-*value *<* 0.0001) and 0.146 (*p-*value < 0.0001) in the covariation correlation assays (see Protocol S4 for the detailed method and results). Interestingly, while applying the covariation assay to the sensory neurons (see Protocol S4), their expression signatures showed a significant correlation only with their outgoing synaptic connections (0.432, *p-*value < 0.0001). This may arise because of the absence of data from the sensory receptors, their main input sources (the connectivity data includes only connections within the neurons).

**Figure 1:**
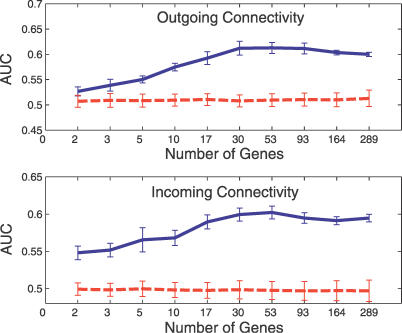
Prediction of Synaptic Connectivity Signatures as a Function of the Most Informative Genes The accuracy of the predictor as a function of the number of genes selected for the predictor is described by the blue line. Prediction accuracy is measured by AUC. The top panel shows the outgoing connectivity results, and the lower panel shows the incoming connectivity results. The rightmost point (289 genes) denotes the prediction outcome before any feature selection is applied to the data. The blue line represents 5-fold cross-validation repetitions of the selection–prediction scheme (mean and standard deviations are displayed). The red dashed lines represent the empirical null hypothesis distribution of performing the selection–prediction scheme on random data (constructed by shuffling the identities of the neurons, see Materials and Methods). Maximum AUC measurements are achieved with 53 and 30 features in the incoming and outgoing assays, respectively, with corresponding *p*-values of *p* = 10^−99^ and *p* = 10^−97^, calculated by applying a one-sided *t*-test between the original and shuffled data (see Materials and Methods).

**Figure 2:**
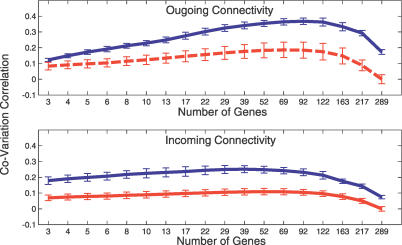
Covariation Correlation Feature Selection Assay The mean and standard deviation of the Pearson correlation (blue line) between the neurons' neighborhood relations in the expression and connectivity spaces is displayed as a function of the number of genes used to determine the expression signature (results of ten repetitions of the assay each with 90% of the neurons). The top panel shows the outgoing connectivity results, and the lower panel shows the incoming connectivity results. The rightmost point (289 genes) denotes the correlation before any feature selection is applied to the data. The dashed red line represents the empirical null hypothesis distribution of the covariation correlation on random data (constructed by shuffling 1,000 times the identities of the neurons and reapplying the analysis to the shuffled data). Maximum correlation measurements are achieved with 39 and 92 features in the incoming and outgoing assays, respectively, with corresponding *p*-values of *p* < 0.0001, *p* = 0.004, respectively (see Materials and Methods).

To identify the genes (features) that highly contribute to the connectivity's prediction accuracy and to the expression–connectivity covariation correlation, an extensive feature selection process was performed (see Materials and Methods). These feature selection assays do not necessarily testify to causal and direct relations but do give rise to putative gene candidates for future experimental investigation studies. Table S2 lists the genes selected in the prediction feature selection assays. Focusing on gene sets that provide the highest AUC performance in each of the connectivity prediction assays resulted in 53 genes that yielded a predictor with an average AUC of 0.60 (*p-*value = 10^−99^) for the incoming connections and 30 genes that yielded a predictor with an average AUC of 0.61 (*p-*value = 10^−97^) for the outgoing connections, as shown in Figure 1 *.* Results of the covariation feature selection assay are shown in Figure 2. As the feature selection process used in the correlation covariation assay is greedy, the procedure for feature selection using correlation covariation is repeated ten times, each repetition applying the assay to a random set composed of 90% of the neurons (see Materials and Methods). Figure 2 presents the mean and standard deviations of these repetitions. The assay results in statistically significant feature sets, leading to a correlation of 0.252 (*p-*value < 0.0001) between expression and incoming connectivity signatures and 0.368 (*p-*value = 0.004) with the outgoing connectivity signatures (*p*-value calculations are described in Materials and Methods)*.* The final gene sets of the covariation assay (listed in Table S3) are produced by focusing only on the genes selected in all ten repetitions of this assay. The latter results in sets of 12 genes for the incoming connectivity (*p-*value *=* 0.04) and 52 genes for the outgoing connectivity (*p-*value *=* 0.02). Evidently, the correlation obtained with respect to the outgoing signatures remains above that obtained with respect to the incoming signatures, testifying that the expression signatures in hand carry more information about outgoing synaptic patterns than about incoming ones.

The outcome of the feature selection process is a list of genes that bear significant information about the specific targets and sources of neuronal synaptic connectivity. Four such sets, generated by the two connectivity types using the two assays, were obtained. To compare these gene lists with contemporary knowledge, we compiled a list of genes known to be involved in neuronal connectivity in C. elegans (see Table 1—these genes are typically involved in axonogenesis and synaptogenesis specificity). All four sets of genes selected in our analysis show a statistically significant overlap with this list of currently known genes: both for the incoming connectivity (*p-*value = 0.005 and *p-*value = 0.026 in the prediction and covariation assays, respectively, using a hypergeometric significance test), and for the outgoing connectivity (*p-*value = 0.021 and *p-*value = 0.018 in the prediction and covariation assays, respectively). The end result of this analysis are two connectivity-specific joint gene sets (Table S4): genes that appear in both types of outgoing assays (11 genes: *ceh-23, che-3, gpa-3, kin-29, kvs-1, lin-11, osm-3, osm-9, tax-2, tax-4,* and *unc-5*) and genes that appear in both types of incoming assays (5 genes: *che-2, mgl-2, mps-1, pef-1,*and *unc-5*).

**Table 1:**
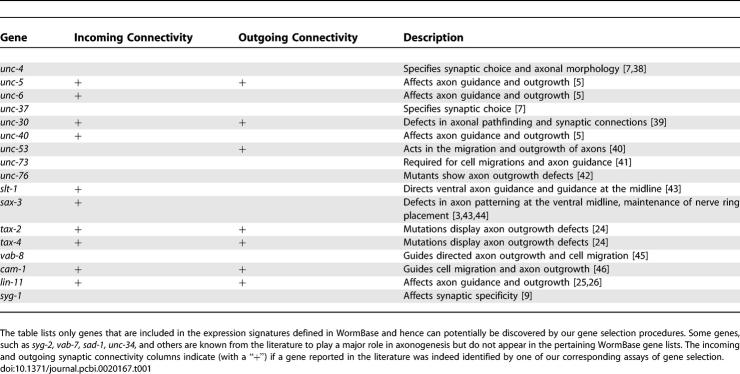
List of Genes Involved in the Analysis Which Have Previously Been Reported in the Literature as Acting in Axonogenesis and Synaptogenesis

## Discussion

Both the gene expression and the neuronal connectivity data gathered from the public databases is obviously not optimal for comprehensively addressing the challenges raised in this study, as it is quite crude and noisy. The crudeness is at least partially due to the usage of a discrete, binary description of the data in hand, while in reality both the expression and connectivity signatures have continuous values. On the connectivity side, these continuous values denote synaptic efficacies, which, together with information on the inhibitory and excitatory function of the synapses, obviously compose very relevant and important “missing information.” On the genetic side, ideally, single neuron genomic expression data collected at different time points during axonal growth and synaptic development should be examined. Indeed, first steps in obtaining more refined data are now being performed [22,23]. Yet, within the limitations of the currently existing large-scale data we successfully identify statistically significant information characterizing the fundamental relation between the expression and synaptic properties of neurons. Our estimations of the predictive information that resides in the neuronal expression data about their connectivity should hence be regarded as rough lower bounds on the true values of this information, given the noisy quality of the data. Yet, there are probably other factors, beyond the genetic properties of the neurons, that determine the synaptic patterns. Such factors may include self-organization mechanisms, distance between neurons, and other cellular properties.

The gene sets identified in the current study are putative candidates for playing a key role in determining and maintaining the synaptic connectivity structure of *C. elegans,* carrying the highest level of information about the connectivity signatures. The list of 15 genes described in Table S4 (results of the intersection between the prediction and covariation correlation assays) compose our most plausible gene targets for further investigation. Indeed, some of the genes in this list were already identified in previous synaptogenesis studies, such as *unc-5, tax-2, tax-4* [24], and *lin-11* [25,26]. Yet, some interesting clues indirectly point to the additional involvement of genes from our list that have not been previously known to be directly involved in synaptogenesis: though there is currently no evidence that *ceh-23* plays a direct role in axon guidance in *C. elegans,* although it does play a role in specific cell differentiation, its Drosophila melanogaster homolog, *dhb-9,* is known to be involved in neural development, axonal pathfinding, and target recognition [27,28]. Even though *mps-1* and *kvs-1* have not been directly associated with axon guidance and development, they have been previously reported as causes of neuronal defects and dysfunction after their inactivation in RNA interference experiments [29]. Some genes on the list are also likely to play a part in axon guidance, targeting, and development due to the processes to which they are annotated; such are genes encoding for G proteins involved in signal transduction (GPA-3) or proteins expressed in the cilia of ciliated neurons (CHE-2, CHE-3). Interestingly, some genes on the list are known to act as specific neuron type identifiers *(osm-9, osm-3)*—hence the information they are carrying regarding the connectivity signatures is probably mediated via their effects on determining cellular fate. Finally, *mgl-2,* identified as specific to the incoming synaptic signature, has a human homolog, *grm-1,* which is known to function as a postsynaptic metabotropic G protein-coupled receptor [30]—this is in line with its appearance solely in the incoming assay list, and supports its role in axon development and regulation.

The relations between the expression and connectivity signatures of neurons and their actual behavior and functional contributions are obviously highly complex and transcend many levels [31]. Previous studies have shown a correlation between the neuronal transcriptome and the electrophysiological phenotypes of neurons, and have shown that one can build a predictor from the former to the latter [32]. But the link from these electrophysiological properties to the neuron's actual functional contribution has been missing. We suggest a new way of addressing this challenge in a rigorous quantitative manner by adding to our analysis an additional signature; the functional contribution signature for each neuron. The latter functional “neuron contribution signature” we refer to can be obtained in our case via a multi-perturbation analysis (MPA) [33] (Protocol S5) of neural laser ablation data published by Bargmann and Horvitz [34]*.* This analysis is described in detail in Protocol S6. The results, alas, are not statistically significant after correcting for multiple hypothesis testing. However, we believe that the approach outlined is of interest, and, with additional data, may lead to significant findings.

This study addresses the relation between neuronal expression and connectivity properties in a large-scale quantitative manner. Though existing small-scale studies have provided ample support for the idea that connectivity has a significant basis in gene expression, the current study rigorously quantifies and measures this relation, providing a “lower bound” estimate on its magnitude. Despite the rough and low precision data available, the results presented and the genes identified provide a promising starting point for further, more detailed computational and experimental investigations. The use of DNA microarrays with hundreds or thousands of simultaneously measured mRNAs, along with a more elaborate description of the neuronal connectivity, should further facilitate our understanding of the relationship between neuronal gene expression and connectivity. Studies similar to the current one would be required to determine whether the findings presented here appear in a variety of other species, using genetic and connectivity information that is gradually being identified, e.g., in cats [35] and humans [36].

## Materials and Methods

### The data.

The neurons expression signatures were obtained from the public WormBase database (http://wormbase.org version WS140), which lists for each neuron the genes with expression patterns directly associated with it (a gene is directly associated with a neuron in the database if the neuron's name is precisely mentioned in relation to the gene in the pertaining expression pattern assays deposited in the database). The data includes 181 neurons (out of the 302 C. elegans neurons), each having at least one gene associated with it. To avoid a bias caused by the symmetric structure of the data, we focused on 98 neurons (only neurons on the right side of the nematode and neurons without a symmetrical companion, Table S1). The resulting *expression signature* of each neuron is a binary vector of 289 genes (see gene list in Table S6), coded as one if the corresponding gene appears to be associated to the neuron and as zero otherwise. Neurons' *connectivity signatures* were obtained from the C. elegans synaptic wiring diagrams, formed by serial sections electron microscopic reconstructions; R. M. Durbin (http://elegans.swmed.edu/parts/neurodata.txt) based on the classic work by White et al. [17] and Hall et al. [18]. We focus on chemical synapses, in which the identities of the presynaptic and postsynaptic neurons are well-defined. Any two neurons may be connected or not with a direction assigned to their connection. Each neuron is thus described by two binary vectors characterizing its connectivity, one for the outgoing synaptic connections (the synapses sent out by the axon of the respective neuron) and one for the incoming synaptic connections (the synapses impinging on it).

### The neuron type classification.

Each of the 98 neurons analyzed were assigned to one or more neuron types according to the WormBase database. The neuron types are: sensory, amphid (including amphid interneurons), cord, motor, ring, labial, and interneurons. See Table S1 for the classification of the neurons.

### The prediction assay.

Prediction was performed using a standard weighted multiclass KNN algorithm, using Euclidean distance between the neurons in the input expression signatures' space. As a preprocessing stage we eliminated features (genes) that were shared by no more than one neuron. The prediction targets, given an input neuron, were its synaptic connections (incoming and outgoing, separately) to all other neurons (each represented as a class in the multiclass prediction). The prediction model's performance score was based on 5-fold cross-validation (training on 80% of the neurons and testing on the resulting 20%). Prediction accuracy was measured by the average area under receiver operating characteristic (ROC) curve (AUC), where averaging was performed over all output classes. The ROC curve plots the fraction of true positives versus the fraction of true negatives for a binary classifier system, while its discrimination threshold varies. AUC is a measure that intuitively can be described as the probability that when randomly picking one positive and one negative example, the classifier will assign a higher score to the positive example than to the negative. AUC can vary from 0 to 1, and, typically, a random prediction will result in an AUC of approximately 0.5. The cross-validation was further used for finding the optimal value of K, the single hyperparameter of KNN*.* Statistical significance of the prediction performance was calculated against an empirical null hypothesis, constructed by repeating the prediction procedure with shuffling: on each such repetition the neuron signatures were shuffled amongst all neurons (that is, shuffling the neuron labels—thus eliminating any functional relation between a neuron and its corresponding signatures while preserving the actual distribution of signatures). To calculate significance levels a one-sided *t*-test was applied, comparing the mean result achieved in the 5-fold cross-validation of the actual data with the empirical distribution achieved with the shuffling. The *t*-test requires data generated from a normal distribution; this assumption was verified by analyzing the quantile–quantile (Q–Q) plots of the empirical distribution. The Q–Q plot graphically compares the percentiles of the distribution of a given variable with that of the normal distribution so that a variable that is normally distributed produces a straight line.

### The covariation correlation assay.

To examine the correlation between two signatures across all neurons under investigation, we used an assay similar to the one used by Toledo–Rodriguez et al*.* [32]*.* Given a set of *N* neurons, where each has two signatures, *s_1_* and *s_2_,* we constructed two *N × N* similarity matrices, *S_1_* and *S_2_*, where *S_1_* (*S_2_*) represents the pairwise similarity between the *s_1_* (*s_2_*) signatures of the neurons. The similarity measure we use is the 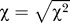 index (Protocol S1 shows the results using alternative similarity measures and explains the reasoning for choosing the χ index)*.* The (N * N / 2 − N) entries forming the lower triangle of *S_1_* (*S_2_*) are concatenated to form a covariation vector *v_1_* (*v_2_*)*.* The Pearson correlation between the two covariation vectors *v_1_* and *v_2_* describes the extent to which the neighborhood relations of the neurons in the two signature spaces *s_1_* and *s_2_* are similar. The statistical significance of the resulting correlation is computed using an empiric null hypothesis constructed from repeating the procedure with shuffling. On each repetition the neuron signatures were shuffled amongst all neurons (shuffling the neuron labels as described above in the prediction assay). To calculate *p*-values we repeated the shuffling 1,000 times and computed the probability to achieve a score equal or higher than the score of the original (nonshuffled) data. This methodology was chosen since, in contrast to the prediction assay, the scores obtained by the shuffling procedure were not normally distributed.

### Feature selection—Prediction assay.

Feature selection was used to find a small subset of genes that yield high accuracy prediction (at least as equally good as that obtained with all the features). A filtering feature selection method was used, ranking the features according to their average mutual information with respect to the multiclass targets [37]. For various sizes, *x,* of feature sets (*x* = 2, 3, 5, 10, 17, 30, 53, 93, 164, 289, taking the first *x* features in the ranked list), the average AUC performance achieved by KNN (similar procedure as in the general case) was calculated (note that the ranking of the features and selecting the optimal K were performed only on part of the data, and measuring the actual performance measure was done on a validation set, not available to the training stages). Statistical significance was computed for the feature set with the highest AUC. The significance level was calculated against an empirical null hypothesis constructed from repeating the feature selection procedure with the same shuffling procedure described above in the “prediction without selection” case, and applying the same *t*-test. (Q–Q plots verify a normal distribution which has permitted us to perform the *t*-test)

### Feature selection—Covariation correlation assay.

Here we used a greedy backward elimination algorithm [37], starting from the complete gene set and iterating while eliminating genes via a greedy algorithm that maximized the correlation covariation measure. In each iteration, 25% of the features (genes) were eliminated according to their marginal influence on the covariation correlation measure when excluded. The feature selection process was repeated ten times, each utilizing 90% of the data, to avoid overfitting and local minima. The selected set of genes used throughout this paper includes only genes that were selected in all ten repetitions of the feature selection process. The statistical significance of the outcome was calculated against an empiric null hypothesis constructed from repeating the identical feature selection procedure with a shuffling procedure as described above, for 1,000 times. The *p*-values for testing the statistical significance of the optimal feature set were computed as follows: for each number of features, *j,* we calculated the mean correlation achieved when applying the feature selection to the shuffled data *C_j_^null^* and its standard deviation *S_j_^null^* (this forms a null hypothesis empirical distribution). For the correlation achieved by the maximum chosen set, C_j*_^true^ (on the actual nonshuffled data), with j* being the number of features in the chosen set, we calculated its variation from the null model Δ = (C_j*_^true^−C_j*_^null^)/ S_j*_^null^. The *p*-value was the probability of achieving such a variation, Δ (or larger) in any of the 1,000 shuffled repetitions with any number of features (for each shuffle we considered the optimal number of features maximizing the variation from the null model). Hence, if significant it testifies that the probability of achieving such a variation by chance (no matter with how many features) is low. The significance level of the size of the gene set chosen (those selected in all ten repetitions) was computed versus the probability of achieving similar sizes or larger when applying an identical procedure to randomly shuffled data.

### Hypergeometric enrichment test.

Given are N genes, where D of them are related to synaptogenesis. Utilizing the hypergeometric distribution, we computed the probability of selecting a sample of *n* genes for which at least k genes were related to synaptogenesis.

## Supporting Information

Protocol S1 Alternative Similarity Measures in the Covariation Analysis Assay (46 KB PDF)

Protocol S2 Individual Neuron Connectivity Prediction (25 KB PDF)

Protocol S3 A Semiparametric Statistical Model of Neuronal Expression–Connectivity Prediction Accuracy (67 KB PDF)

Protocol S4 Quantifying the Relation Between Expression and Connectivity Signatures while Controlling for Neuron Type (27 KB PDF)

Protocol S5 Multi-Perturbation Shapley Value Analysis of Neuronal Role in Chemotaxis (28 KB PDF)

Protocol S6 Relation Between Expression and Connectivity Signatures to the Functional Contributions of Neurons (27 KB PDF)

Table S1 List of Neurons and Each Neuron's Type (169 KB DOC)

Table S2 List of Genes Selected in the Prediction Feature Selection Assay (20 KB XLS)

Table S3 List of Genes Selected in the Covariation Correlation Feature Selection Assay (16 KB XLS)

Table S4 List of Genes Selected in Both Prediction and Covariation Correlation Assays (18 KB XLS)

Table S5 Chemotaxis Neuron Contribution Signatures (15 KB XLS)

Table S6 List of Genes Part of the Analysis (25 KB XLS)

## Abbreviations

AUC: area under the receiver operating characteristic curve
KNN: k–nearest neighbor prediction algorithm
ROC: receiver operating characteristic
