# Supplementary material for: Gene Expression of Caenorhabditis elegans Neurons Carries Information on Their Synaptic Connectivity
Source: PLoS Comput Biol. 2006 Dec 8;2(12):e167. doi: 10.1371/journal.pcbi.0020167 (PMC1676027; doi:10.1371/journal.pcbi.0020167)
Supplement: Supplementary file 1 [file pcbi.0020167.sd001.pdf]

Protocol S1

Alternative Similarity Measures in the Covariation Analysis Assay

The covariation correlation assay examines the correlation between two signature spaces across all neurons. Essentially, the assay compares to what extent are two similarity matrices (each for a different kind of signature, e.g., expression and connectivity) correlated. In the main text the square root of the χ^2 index (χ) is utilized to compute the similarity/distance matrix in the expression and connectivity spaces. We then further compare the two similarity matrices by computing the Pearson correlation between the two. Here we provide the results of using alternative similarity measures in this procedure, and provide the reasoning for choosing the χ index.

Each of the two pairwise similarity matrices is calculated from the given binary vectors as follows: Given two vectors *x* and *y* with a length of *n* features, the similarity between the two is computed from a 2*2 contingency table, which for each pair of neurons describes the number of cases in which they "agree" or "disagree". In a more formal definition, let:

- $a = sum(\sim x \& \sim y)$, be the cases in which the feature is absent from both neurons.
- $b = sum(x \& \sim y)$, be the cases in which neuron *a* has the feature while neuron *b* does not.
- $c = sum(\sim x \& y)$, be the cases in which neuron *b* has the feature while neuron *a* does not.
- $d = sum(x \& y)$, be the cases in which the feature exists in both neurons.
- $n = a + b + c + d$.

The similarity measures we examined can be described in these terms as follows:

$$\chi^2 = \frac{n(ad - cb)^2}{(a+b)(c+d)(a+c)(b+d)}$$

$$\chi = \sqrt{\frac{n}{(a+b)(c+d)(a+c)(b+d)}}\,|ad-cb|$$

Pearson coefficient = $\dfrac{ad-cb}{\sqrt{(a+b)(c+d)(a+c)(b+d)}}$

Yule's coefficient = $\dfrac{ad-cb}{ad+cb}$

Jaccard similarity coefficient = $\dfrac{d}{b+c+d}$

Applying these measures (using absolute values) shows qualitatively similar results, although the numerical values vary. The table below shows the correlation between the similarity matrix of the expression signature to the similarity matrix of the connectivity signature using the different similarity measures (incoming and outgoing connectivity signatures). p-values are calculated with respect to an empirical background model calculated by reapplying the assay 10000 times to randomly shuffled data (see methods):

	Incoming Connectivity	Outgoing Connectivity
χ^2	0.140 ($p<10^{-4}$)	0.215 ($p<10^{-4}$)
χ	0.075 ($p<10^{-4}$)	0.176 ($p<10^{-4}$)
Pearson correlation	0.075 ($p<10^{-4}$)	0.176 ($p<10^{-4}$)
Yule's coefficient	0.103 ($p=0.002$)	0.102 ($p=0.002$)
Jaccard	0.147 ($p<10^{-4}$)	0.184 ($p<10^{-4}$)

Although the precise genetic expression/connectivity association levels differ from one measure to another, the significance levels remain similar. The use of the χ index throughout the main text was chosen due to its statistical advantages as explained in the following. Evidently, the three measures we compare, χ^2, Pearson coefficient and the Yule coefficient all have the same numerator, ($ad-bc$, the determinant of the contingency table) with different normalization factors as denominator. The χ^2 statistic has an approximate χ^2 distribution with one degree of freedom, this approximation being almost exact regardless of *a,b,c,d* and *n*, as long as

$\frac{1}{n}\min(a+b,c+d)\min(a+c,b+d)\geq 5$. Even if not all cases satisfy this condition, a nearly scenario-free statistic is preferable. Thus, these χ^2 measures, computed from diverse contingency tables (as we do when we correlate the similarity matrices) come from the same baseline distribution, corresponding to the case in which the null hypothesis of randomness is always correct.

The Yule statistic, on the other hand is the product of the χ^2 measure by a contingency–table–dependent normalization term which introduces additional noise: when aggregating diverse contingency tables, using the Yule coefficient results in an unnecessarily more dispersed distribution, so the detection of signal becomes weaker, with bigger p-values. In statistical terms, the test based on Yule has smaller power than the test based on χ^2. Since we further compute the Pearson correlation between the resulting similarity matrices, an index with a normal-type distribution would be preferable, and hence $\chi=\sqrt{\chi^2}$, which has an (absolute) normal distribution, would be a proper choice. As it turns out for the binary case, this measure is equal to $\sqrt{n} * |\text{Pearson coefficient}|$.
