# Supplementary material for: Gene Expression of Caenorhabditis elegans Neurons Carries Information on Their Synaptic Connectivity
Source: PLoS Comput Biol. 2006 Dec 8;2(12):e167. doi: 10.1371/journal.pcbi.0020167 (PMC1676027; doi:10.1371/journal.pcbi.0020167)
Supplement: Supplementary file 2 [file pcbi.0020167.sd002.pdf]

Protocol S2

Individual Neuron Connectivity Prediction

In the main text we report averaged prediction level across all neurons. The average AUC obtained shows highly significant results yet rather moderate levels of AUC: For the incoming connectivity signatures the average AUC is 0.594 (*p-value* $=10^{-85}$) and for the outgoing connectivity signatures the average AUC is 0.601 (*p-value* $=10^{-75}$) (see main text for details). One putative cause of the moderate levels found may arise since the prediction levels reported are averaged across all neurons. To examine this further we applied an additional set of analysis assays aimed at identifying subsets of neurons manifesting high levels of association between their genetic and connectivity signatures.

To identify the accuracy of predicting the incoming and outgoing connectivity of each neuron individually, the area under ROC curve (AUC) is computed for each neuronal connectivity predictor and its statistical significance is tested (see Methods in the main text for a detailed description of the prediction assay). The significance level of the AUC measured per neuron, is computed empirically by repeating the same procedure 10,000 times with shuffled expression data, and computing the probability to obtain AUC levels which are similar or higher than the non-shuffled data. We correct for multiple comparisons (there is a single hypothesis for each neuron) using false discovery rate (FDR) of 0.05.

Applying the assay to the incoming connectivity signatures identifies 24 neurons with statistically significant prediction levels (out of the set of 132 neurons), 15 for which the AUC values are above 0.594 and reach AUC=0.95. Similarly, 25 neurons with statistically significant prediction levels were identified in the outgoing synaptic connectivity case (out of 116), for 15 of which the AUC values are above 0.601 and reach AUC=0.98. Hence, we find and identify quite a few neurons for which the ability to predict their connectivity is high and significant, even though the average AUC is rather moderate.

The list of neurons manifesting AUC levels above the average level are as follows, with corresponding AUC values and p-values before FDR correction:

Incoming connectivity neurons:

Neuron	AUC	p-value
AVJR	0.950	<0.001
PVPL	0.884	<0.001
AVFL	0.880	<0.001
RIAR	0.880	<0.001
AIAL	0.865	0.008
ASIL	0.865	0.008
RIAL	0.860	0.002
RIML	0.848	<0.001
OLLL	0.845	0.006
DVA	0.790	0.002
RIH	0.754	<0.001
AIAR	0.705	<0.001
RMDL	0.683	<0.001
AIZR	0.625	<0.001
RIPR	0.613	0.002

Outgoing connectivity neurons:

Neuron	AUC	p-value
AWBR	0.986	<0.001
AIYR	0.979	<0.001
AVDL	0.898	<0.001
RIPR	0.891	<0.001
AIAR	0.883	<0.001
AIAL	0.865	0.008
AIYL	0.865	0.008
OLQVR	0.847	0.002
BDUR	0.842	<0.001
RMHL	0.824	0.006
OLLR	0.823	0.002
PVCR	0.821	<0.001
ADER	0.713	0.010
IL2R	0.685	0.002

RICL	0.622	0.002

Neuron names according to the WormBase database (http://wormbase.org).
