# Supplementary material for: Gene Expression of Caenorhabditis elegans Neurons Carries Information on Their Synaptic Connectivity
Source: PLoS Comput Biol. 2006 Dec 8;2(12):e167. doi: 10.1371/journal.pcbi.0020167 (PMC1676027; doi:10.1371/journal.pcbi.0020167)
Supplement: Supplementary file 3 [file pcbi.0020167.sd003.pdf]

Protocol S3

A Semi Parametric Statistical Model of Neuronal Expression-Connectivity Prediction Accuracy

Evaluating the performance of the predictor assay has been done using classical frequentist inference, as presented in the main text and Protocol S2. Here we complement this evaluation by an additional approach - constructing a statistical semi parametric model of AUC prediction accuracy of each neuron's connectivity from its expression signature. The importance of constructing such a model lies in the ability to reliably estimate the AUC value at the tails of the distribution, in particular, the relatively rare cases which achieve very high accuracy levels, for which there are typically very few samples to infer from (rendering the standard empirical frequency approach useless in such cases). To this end, the semi parametric model outlined below is constructed to faithfully describe the probability of finding neurons with a specific level of AUC.

We describe the distribution of the AUC values obtained for each neuron (predictor-based-distribution (PDB)) in relation to a random benchmark constructed by repeating the prediction assay on data in which the gene expression signatures are shuffled among the neurons, and the connectivity signatures are left intact. Letting $f(x)$ describe the density of neurons with an AUC of x in the shuffled benchmark distribution, we model $g(x)$, the corresponding density of the PDB. A bi-variate exponential type family of distributions with sufficient statistic $u(x), v(x)$ is used, as described in equation (1.1):

(1.1) $$g(x) = f(x)e^{\theta u(x)+\eta v(x)-b(\theta,\eta)}$$

The normalizing constant $b(\theta,\eta)$ makes $g(x)$ a density, i.e., equation (1.2):

(1.2) $$e^{b(\theta,\eta)} = \int e^{\theta u(x)+\eta v(x)} f(x)d(x)$$

Since $f(x)$ is unknown, we replace $f(x)$ by its standard estimate, the empirical distribution of the shuffled sample, based on N observations. This way $b(\theta,\eta)$ is approximated by $\tilde{b}(\theta,\eta)$, given by equation (1.3):

(1.3) $$e^{\tilde{b}(\theta,\eta)} = \frac{1}{N}\sum_{i=1}^{N} e^{\theta u(x)+\eta v(x)}$$

Maximum Likelihood Estimation of the parameters θ and η consists in maximizing the likelihood function (1.4) or its logarithm.

(1.4) $$\prod_{j=1}^{n} f(x_j) e^{\theta\sum_{j=1}^{n} u(x_j)+\eta\sum_{j=1}^{n} v(x_j)-b(\theta,\eta)}$$

Since

(1.5) $$\frac{\partial \log(\prod_{j=1}^{n} f(x_j) e^{\theta u(x_j)+\eta v(x_j)-b(\theta,\eta)})}{\partial\theta} = \sum_{j=1}^{n} u(x_j) - n\frac{\partial b(\theta,\eta)}{\partial\theta}$$

and

(1.6) $$\frac{\partial \log(\prod_{j=1}^{n} f(x_j) e^{\theta u(x_j)+\eta v(x_j)-b(\theta,\eta)})}{\partial\eta} = \sum_{j=1}^{n} v(x_j) - n\frac{\partial b(\theta,\eta)}{\partial\eta}$$

we get the usual exponential-type MLE, that fits θ and η by equating the theoretical means $\frac{\partial b(\theta,\eta)}{\partial\theta}$, $\frac{\partial b(\theta,\eta)}{\partial\eta}$ of the sufficient statistics $u(x), v(x)$ to their empirical means $\frac{1}{n}\sum_{j=1}^{n} u(x_j)$ and $\frac{1}{n}\sum_{j=1}^{n} v(x_j)$. For this procedure, $\tilde{b}(\theta,\eta)$ plays the role of $b(\theta,\eta)$. Confidence (rays or) intervals for θ and η can be obtained from the Fisher information (FI) matrix, the matrix FI of second derivatives of $b(\theta,\eta)$: the standard deviation of θ (respectively η) is estimated by $\sqrt{FI^{-1}(1,1)}$ ($\sqrt{FI^{-1}(2,2)}$).

Choice of $u(x), v(x)$: We could consider just one term, with linear (x) or logit, $\log(\frac{x}{1-x})$, statistic u. Logit is the most commonly used transformation for frequency data, and it gave a reasonable fit. However, the value x=1 occurs with some small positive frequency, so x would have to be artificially and arbitrarily truncated at some value close to 1.

Instead, we opted for the simplest polynomial transformation that preserves the anti-symmetric nature of the logit function, namely, $\theta(x-0.5)+\eta(x-0.5)^3$. This family lets data speak for itself by including linear behavior, logit-like behavior as well as S-shaped behavior. We thus take $u(x)=x-0.5$ and $v(x)=(x-0.5)^3$.

Applying this semi-parametric model construction approach to our neuronal AUC prediction data shows a nice fit: Figure 1 presents the results of applying it to the prediction accuracies of incoming connectivity (the estimated parameters are $\hat{\theta}$=4.92 (st error=0.278), $\hat{\eta}$=-5.715 (st error=1.804) and $\tilde{b}(\theta,\eta)$=-0.016, significantly manifesting S-shaped behavior). Figure 2 describes the results when applied to the prediction accuracy levels of outgoing connectivity data (the estimated parameters are $\hat{\theta}$=3.166 (st error=0.31), $\hat{\eta}$=7.569 (st error=1.835) and $\tilde{b}(\theta,\eta)$=0.028, displaying significant logit-like behavior). These figures show the empirical distribution obtained with random shuffled data in blue, the empirical AUC results obtained with the prediction assay in red (PBD), and the semi-parametric model's predictions in green (with a 95% confidence band in dashed green lines). The model fits the data reasonably well, as is also evident from the adequate Kolmogorov-Smirnov test results obtained. Further evidence to the model's good fit arises from examining the empirical 2nd order moment of x (as the 1st and 3rd order moments are preset in the computation outlined above), which is identical to the model predicted one in the first three significant decimal digits.

Thus, this model provides us with a reliable tool to discriminate between AUC values obtained from the PDB and the random distribution. In particular, this is of importance for the relatively rare cases achieving very high accuracy levels. The model exhibits a specific pattern of the stochastic dominance of the baseline distribution by the PBD in which discrimination between the two amplifies at high AUC levels to sizable probability ratios. Furthermore, this finding establishes (and quantifies) AUC as a meaningful measure of predictability of connectivity from gene expression. The actual existence of neurons that manifest high AUC values is described in Protocol S2. Based on the model, once a predictor's AUC is given, one can compute the ratio between the probabilities to achieve such an AUC from the PBD and from a random predictor. For

example, for AUC values of *0.9* the ratio is *5.05* in the incoming connectivity case and *5.59* in the outgoing case.

Application of this modeled probability ratio, possibly in a Bayesian framework, is a subject for further research, we defer from actually taking this step further in this paper.

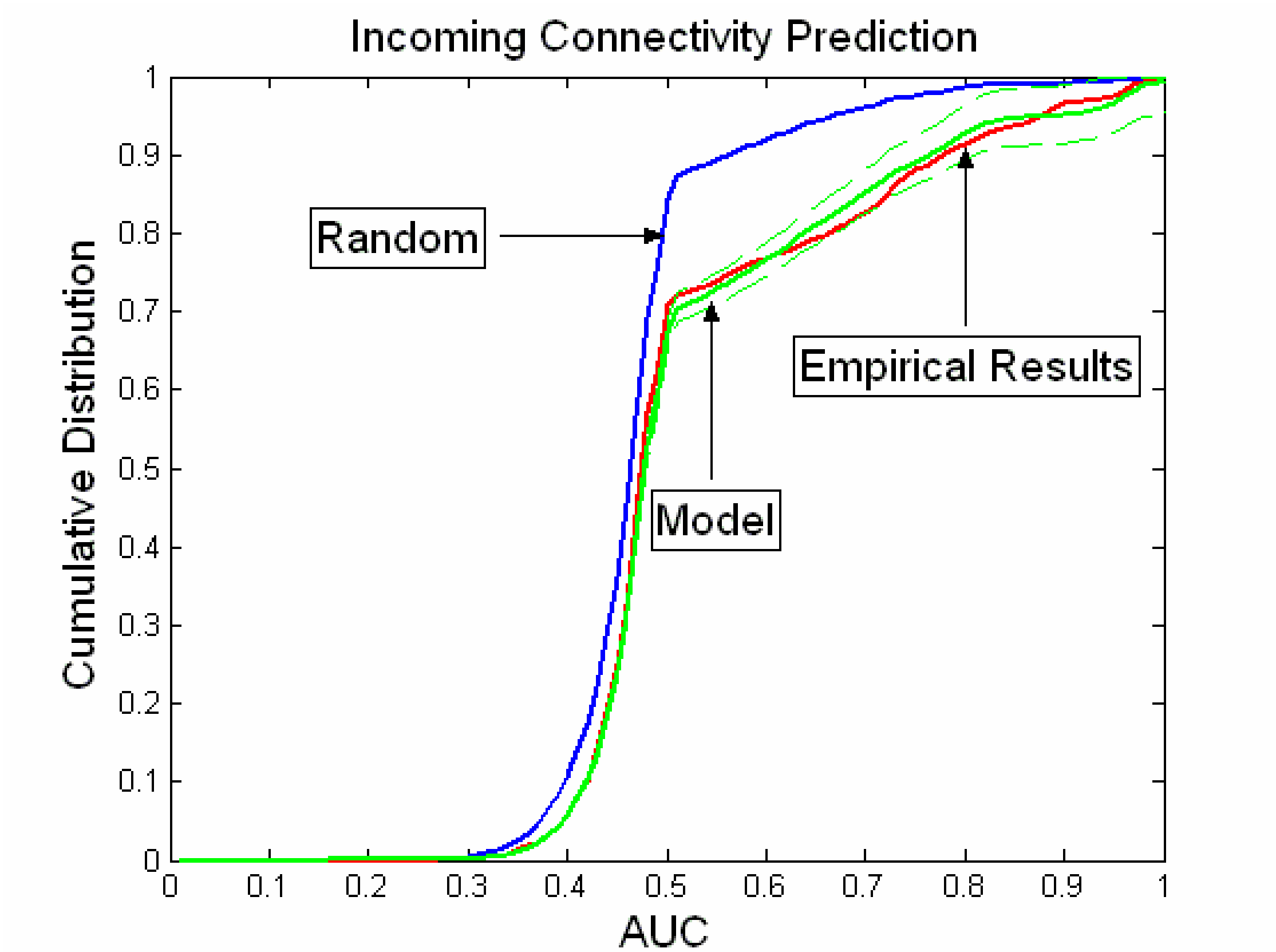

Figure 1: Model evaluation in the incoming connectivity assay. The cumulative distribution function is plotted against the prediction performance, AUC. The blue line represents the empirical random AUC distribution, the red line the actual empirical AUC achieved and the green line the models fit to the data with a confidence band of 95% (dash green).

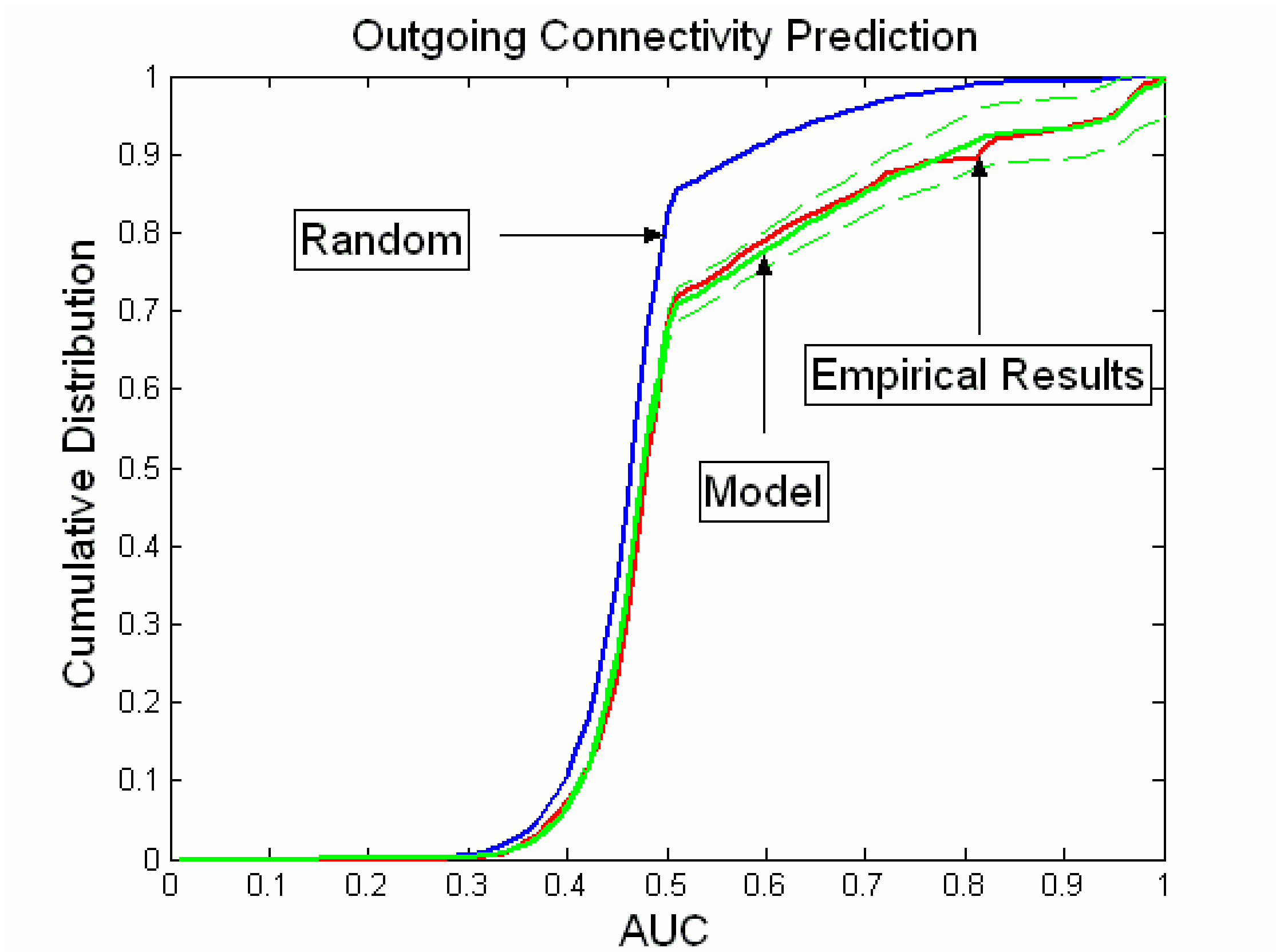

Figure 2: Model evaluation in the outgoing connectivity assay. The cumulative distribution function is plotted against the prediction performance, AUC. The blue line represents the empirical random AUC distribution, the red line the actual empirical AUC achieved and the green line the models fit to the data with a confidence band of 95% (dash green).
