# Supplementary material for: Gene Expression of Caenorhabditis elegans Neurons Carries Information on Their Synaptic Connectivity
Source: PLoS Comput Biol. 2006 Dec 8;2(12):e167. doi: 10.1371/journal.pcbi.0020167 (PMC1676027; doi:10.1371/journal.pcbi.0020167)
Supplement: Supplementary file 4 [file pcbi.0020167.sd004.pdf]

Protocol S4

Quantifying the Relation between Expression and Connectivity Signatures While Controlling for Neuron Type

The relation between the expression and connectivity signatures reported in the main text may potentially be explained by complete intermediary mediation of the neuron type; that is, expression signatures may determine the neuron type, and following, the later may potentially share similar connectivity signatures. However, a course inspection shows that the variability between connectivity signatures within each neuron type remains of the same order of magnitude as the variability of connectivity measured over all neurons. Hence neuron type per se does not reduce the variability of neuronal connectivity. Furthermore, additional analysis shows that the relation between expression and connectivity signatures exists within neuron types as well.

To this end, we use the same prediction and correlation co-variation assays presented in the main text, measuring the level of prediction and correlation, and test its significance versus a background shuffling model. However, we now shuffle neurons only within their specific neuron type group. Finding a statistically significant relation with this background model would imply that the relation between the expression and connectivity signatures exists also within each neuron type. The analysis is performed on 5 distinct neuron types (for neuron types see Table S1), overall composed of 83 neurons:

1. The sensory neurons.
2. The labial neurons.
3. Motor neurons which are not defined as interneurons as well.
4. Interneurons which are not defined as motor neurons as well.
5. Neurons defined as motor and interneurons.

Table 1 below shows that the relation remains significant even when the effect of neuron type is controlled for.

Prediction assay		Co-Variation correlation	
Incoming	Outgoing	Incoming	Outgoing
0.599($p=10^{-67}$)	0.611($p=10^{-59}$)	0.089($p<0.0001$)	0.146($p<0.0001$)

Table 1: Prediction and co-variation correlation measures in each of the assays, with the significance level based on shuffling neurons within each of the 5 distinct neuron types (the measures are average AUC in the prediction assay and Pearson correlation in the co-variation assay, p-values are calculated as explained in the main text).

One would ideally want to perform a prediction analysis to the neurons belonging to each neuron type separately. Unfortunately this is currently impossible since the number of neurons in each type is too small for a reliable prediction assay. Applying the co-variation correlation assay is more useful in this case, as shown in Table 2 below. Not all cases yield a significant correlation, yet in most neuron types we do find such a relation.

	Incoming	Outgoing
Sensory	0.164 ($p=0.210$)	0.432 ($p<10^{4}$) *
Cord	0.071 ($p=0.681$)	0.311 ($p=0.239$)
Amphid	0.197 ($p=0.047$)	0.300 ($p=0.002$) *
Motor	0.626 ($p=0.012$) *	0.458 ($p=0.014$) *
Ring	0.170 ($p=0.001$) *	0.197 ($p=0.011$) *
Labial	0.720 ($p=0.002$) *	0.571 ($p<10^{4}$) *
Inter	0.109 ($p=0.003$) *	0.148 ($p=0.003$) *

Table 2: Co-variation correlation results between expression signatures and connectivity signatures (incoming / outgoing) within each neuron type. p-values are calculated verse a random shuffling background model, within the 5 distinct neuron types. False discovery rate (FDR) correction was applied for multiple comparisons, * denotes p-values that pass the FDR test with q-value=0.05.

These results demonstrate that the relation we point to is maintained even when controlling for the neuron type. Obviously, the neuron type is significantly determined by the expression signatures (indeed, as shown in the main text using a prediction assay). However, the individual neuronal expression signatures carry significant information about their connectivity when controlling for their neuron types.
