# Supplementary material for: Gene Expression of Caenorhabditis elegans Neurons Carries Information on Their Synaptic Connectivity
Source: PLoS Comput Biol. 2006 Dec 8;2(12):e167. doi: 10.1371/journal.pcbi.0020167 (PMC1676027; doi:10.1371/journal.pcbi.0020167)
Supplement: Supplementary file 5 [file pcbi.0020167.sd005.pdf]

Protocol S5

Multi-Perturbation Shapley Value Analysis of Neuronal Role in Chemotaxis

Perturbation studies, in which functional performance is measured after deletion, mutation or lesion of the different elements, have been traditionally employed to causally deduce the roles played by system elements (neurons, genes, proteins, brain regions, etc.) in performing some function of interest. However, the vast majority of these studies have perturbed only one element at a time, often resulting in little phenotypic effect. In complex biological systems, multiple concomitant perturbations should be employed to reveal the contributions of the different elements to the system's functioning. To this end, the Multi-Perturbation Shapley value Analysis (MPA) method was developed (for a detailed description of the MPA method see[1,2]). The goal of MPA is to compute the contribution (importance) of system elements to a certain function, from a data set of multi-perturbation experiments. In each such experiment, a different subset of the system elements is concomitantly perturbed (denoting a perturbation configuration), and the system's performance in the studied function is measured.

The basic observation underlying MPA is that this set of perturbation configurations is essentially equivalent to a coalitional game in the framework of Game Theory. Hence, borrowing concepts from Game Theory, MPA utilizes the *Shapley value* as the unique fair measure of each element's contribution (importance) to the function in question. The Shapley value has a clear intuitive interpretation denoting the importance of an element to the performance studied averaged over all the possible combinations of subsets of elements in which it can participate. Importantly, it has an axiomatic foundation, which is well suited for the analysis of biological data[1,2]. The end result of MPA analysis is a real number which is assigned to each element, denoting its contribution value (relative importance) to the task studied. The set of contribution values assigned to all the system elements is normalized such that they sum up to one.

In the context of this study, the MPA analysis is used to compute the contribution of each neuron to chemotaxis towards a given attractant. We analyze four different chemotaxis tasks (to four different attractants) and obtain a vector of length four which serves as the contribution signature of each neuron. The analysis is performed on data gathered by neuronal laser ablation experiments probing the chemotaxis behavior of the nematode to various chemical attractants[3]. In these experiments, the nematode directs its movements according to chemical gradients in the environment, moving toward the highest concentration of food or fleeing from toxins. The elements studied are 8 sensory neuron pairs (out of a total of 16 pairs which form the chemosensory system)[4]. In each laser ablation experiment both neurons in a pair are either intact or ablated. The performance measures, chemotaxis to various attractants (each composing a distinct functional task), were evaluated by placing the animal on an agar plate with a gradient of an attractant on one side of the plate, and scoring the chemotaxis performance by counting the number of times the animal arrived at the peak of the gradient minus the number of times the animal arrived at the control plug at the opposite side of the plate. The level of chemotaxis performance was evaluated under 31 perturbation configurations, according to the protocol described in[3]. Prediction of the full, 256 multi-lesion set, which is needed to calculate the neurons' contribution values, was obtained using Projection Pursuit Regression[5] as the predictor. A cross validation leave-one-out procedure shows that the predictor successfully explains 65-80% of variance in the data, depending on the attractant type. Table S5 displays the contributions of the different neuron pairs to the 4 different attractants tasks (Serotonin, Cl, cAMP and Biotin).

REFERENCES

1. Keinan A, Sandbank B, Hilgetag CC, Meilijson I, Ruppin E (2004) Fair Attribution of Functional Contribution in Artificial and Biological Networks. Neural Comp 16: 1887-1915.
2. Kaufman A, Keinan A, Meilijson I, Kupiec M, Ruppin E (2005) Quantitative Analysis of Genetic and Neuronal Multi-Perturbation Experiments. PLoS Computational Biology 1: e64.
3. Bargmann CI, Horvitz HR (1991) Chemosensory neurons with overlapping functions direct chemotaxis to multiple chemicals in C. elegans. Neuron 7: 729-742.

4. Bargmann CI, Mori I (1997) Chemotaxis and thermotaxis In C. elegans II.: Cold Spring Harbor Press. 717-737 p.
5. Friedman JH, Stuetzle W (1981) Projection pursuit regression. Journal of the American Statistical Association 76: 817-823.
