# Supplementary material for: Gene Expression of Caenorhabditis elegans Neurons Carries Information on Their Synaptic Connectivity
Source: PLoS Comput Biol. 2006 Dec 8;2(12):e167. doi: 10.1371/journal.pcbi.0020167 (PMC1676027; doi:10.1371/journal.pcbi.0020167)
Supplement: Supplementary file 6 [file pcbi.0020167.sd006.pdf]

Protocol S6

Relation between Expression and Connectivity Signatures to the Functional Contributions of Neurons.

Extending the realm of the analysis presented in the main text to the functional/behavioral dimension, we turn to examine the relations between their expression and connectivity signatures and the functional contribution of these neurons in a small set of neurons responsible for chemotaxis in the worm. The latter functional “neuron contribution signature” is obtained via Multi-Perturbation Analysis (MPA)[1] of neural laser ablation data published by Bargmann and Horvitz[2] (and reviewed in Protocol S5), focusing on the 8 amphid neuron pairs. Quantifying these relations, we address a classical question in Neuroscience; what dominates the functionality of a neural circuit – the local, genetic basis of the individual neurons, or the overall network structure determined by their connectivity.

The contribution signature of a neuron describes the causal roles (contributions) that a neuron plays during behavior (chemotaxis). Probing the chemotaxis behavior of the nematode to various chemical attractants under a set of multiple neuron ablations, allows one to determine the contribution value of each neuron studied to the task of chemotaxis via MPA. The contribution of each neuron represents its relative importance to successful chemotaxis. It is computed by measuring (or estimating) the decrease in the chemotaxis ability of the nematode when the pertaining neuron is ablated, averaging over all possible multi-knockout configurations (see Protocol S5 for a detailed description of the MPA and its application to the worm chemotaxis data). The contribution computation is repeated for 4 different chemotaxis tasks to 4 different attractants, resulting in a contribution signature for each neuron as a vector of 4 continuous values. For each task, the

contributions of all neurons are normalized such that they all sum up to 1 and reflect relative contributions (see Table S5).

Applying the covariation correlation assay to the chemotaxis contribution data results in insignificant correlations between the expression-contribution and the connectivity-contribution signatures. However, using feature selection identifies subsets of genes and neuronal connections with borderline significance levels before correcting for multiple hypotheses testing (there are four hypotheses), which hence are not significant, but may yet point to some interesting trends. Before correcting for multiple hypotheses, the expression signature shows the highest correlation with the neuronal functional contribution signature (0.880, *p-value* = 0.04). The incoming connectivity signature shows no significant correlation and the outgoing synaptic connectivity does manifest a correlation of 0.603 *(p-value* = 0.05). Accordingly, the outgoing synaptic connections of the amphid neurons seem to be more important for successful chemotaxis than their input connections from other neurons (recall that synaptic inputs from sensory receptors are not included in the connectivity data). Combining both the expression and connectivity signatures to a single joint features signature for each neuron and repeating the assay does not yield any improvement over the correlation obtained using genetic features solely (0.881, *p-value* = 0.06, again, without correcting for multiple hypotheses). These results, if indeed further validated with more data to the extent of statistically significant levels, may testify to the role of the expression properties in determining the behavioral roles of amphid neurons during chemotaxis (at least given the current connectivity data, which lacks information on incoming sensory receptors' connectivity). But a decisive interpretation of such results will remain difficult and hampered by the high correlation between the expression and connectivity signatures which has been demonstrated in the main text.

REFERENCES

1. Kaufman A, Keinan A, Meilijson I, Kupiec M, Ruppin E (2005) Quantitative Analysis of Genetic and Neuronal Multi-Perturbation Experiments. PLoS Computational Biology 1: e64.
2. Bargmann CI, Horvitz HR (1991) Chemosensory neurons with overlapping functions direct chemotaxis to multiple chemicals in C. elegans. Neuron 7: 729-742.
