# Supplementary material for: Gene Expression of Caenorhabditis elegans Neurons Carries Information on Their Synaptic Connectivity
Source: PLoS Comput Biol. 2006 Dec 8;2(12):e167. doi: 10.1371/journal.pcbi.0020167 (PMC1676027; doi:10.1371/journal.pcbi.0020167)
Supplement: Supplementary file 7 [file pcbi.0020167.st001.doc]

Table S1: Neuron List

List of the 98 asymmetric neurons included in the analysis

| Index | Neuron | Sensory^[1]^ | Amphid^[2]^ | Cord^[3]^ | Motor^[4]^ | Ring^[5]^ | Labial^[6]^ | Inter^[7]^ |
| --- | --- | --- | --- | --- | --- | --- | --- | --- |
| 1 | ADAR |  |  |  |  | + |  | + |
| 2 | ADER | + |  |  |  |  |  |  |
| 3 | ADFR | + | + |  |  |  |  |  |
| 4 | ADLR | + | + |  |  |  |  |  |
| 5 | AFDR | + | + |  |  |  |  |  |
| 6 | AIAR |  | + |  |  |  |  | + |
| 7 | AIBR |  | + |  |  |  |  | + |
| 8 | AIMR |  |  |  |  | + |  | + |
| 9 | AINR |  |  |  |  | + |  | + |
| 10 | AIYR |  | + |  |  |  |  | + |
| 11 | AIZR |  | + |  |  |  |  | + |
| 12 | ALA |  |  |  |  |  |  |  |
| 13 | ALMR | + |  |  |  |  |  |  |
| 14 | ALNR |  |  |  |  |  |  |  |
| 15 | AQR |  |  |  |  |  |  |  |
| 16 | ASER | + | + |  |  |  |  |  |
| 17 | ASGR | + | + |  |  |  |  |  |
| 18 | ASHR | + | + |  |  |  |  |  |
| 19 | ASIR | + | + |  |  |  |  |  |
| 20 | ASJR | + | + |  |  |  |  |  |
| 21 | ASKR | + | + |  |  |  |  |  |
| 22 | AUAR | + | + |  |  |  |  |  |
| 23 | AVAR |  |  | + |  |  |  | + |
| 24 | AVBR |  |  | + |  |  |  | + |
| 25 | AVDR |  |  | + |  |  |  | + |
| 26 | AVER |  |  | + |  |  |  | + |
| 27 | AVFR |  |  |  |  |  |  | + |
| 28 | AVHR |  |  |  |  |  |  |  |
| 29 | AVJR |  |  |  |  |  |  |  |
| 30 | AVKR |  |  | + |  | + |  | + |
| 31 | AVL |  |  | + |  | + |  | + |
| 32 | AVM | + |  |  |  |  |  |  |
| 33 | AWAR | + | + |  |  |  |  |  |
| 34 | AWBR | + | + |  |  |  |  |  |
| 35 | AWCR | + | + |  |  |  |  |  |
| 36 | BAGR |  |  |  |  |  |  |  |
| 37 | BDUR |  |  |  |  |  |  |  |
| 38 | CEPDR |  |  |  |  |  |  |  |
| 39 | CEPVR |  |  |  |  |  |  |  |
| 40 | DVA |  |  |  |  | + |  | + |
| 41 | DVB |  |  |  |  | + |  | + |
| 42 | DVC |  |  |  |  | + |  | + |
| 43 | FLPR |  |  |  |  |  |  |  |
| 44 | HSNR |  |  |  |  |  |  |  |
| 45 | IL1DR |  |  |  |  |  | + |  |
| 46 | IL1R |  |  |  |  |  | + |  |
| 47 | IL1VR |  |  |  |  |  | + |  |
| 48 | IL2DR |  |  |  |  |  | + |  |
| 49 | IL2R |  |  |  |  |  | + |  |
| 50 | IL2VR |  |  |  |  |  | + |  |
| 51 | OLLR |  |  |  |  |  | + |  |
| 52 | LQDR |  |  |  |  |  | + |  |
| 53 | LQVR |  |  |  |  |  | + |  |
| 54 | PLNR |  |  |  |  |  |  | + |
| 55 | PVCR |  |  | + |  |  |  | + |
| 56 | PVNR |  |  |  |  |  |  | + |
| 57 | PVPR |  |  |  |  |  |  | + |
| 58 | PVQR |  |  |  |  |  |  | + |
| 59 | PVR |  |  | + |  | + |  | + |
| 60 | RIAR |  |  |  |  | + |  | + |
| 61 | RIBR |  |  |  |  | + |  | + |
| 62 | RICR |  |  |  |  | + |  | + |
| 63 | RID |  |  |  |  | + |  | + |
| 64 | RIFR |  |  |  |  | + |  | + |
| 65 | RIGR |  |  |  |  | + |  | + |
| 66 | RIH |  |  |  |  | + |  | + |
| 67 | RIMR |  |  |  |  | + |  | + |
| 68 | RIPR |  |  |  |  | + |  | + |
| 69 | RIR |  |  |  |  | + |  | + |
| 70 | RIS |  |  |  |  | + |  | + |
| 71 | RIVR |  |  |  |  | + |  | + |
| 72 | RMDDR |  |  |  | + | + |  | + |
| 73 | RMDR |  |  |  | + | + |  | + |
| 74 | RMDVR |  |  |  | + | + |  | + |
| 75 | RMED |  |  |  | + | + |  |  |
| 76 | RMER |  |  |  | + | + |  |  |
| 77 | RMEV |  |  |  | + | + |  |  |
| 78 | RMFR |  |  |  | + | + |  | + |
| 79 | RMGR |  |  |  |  | + |  | + |
| 80 | RMHR |  |  |  | + | + |  | + |
| 81 | SAADR |  |  |  |  | + |  | + |
| 82 | SAAVR |  |  |  |  | + |  | + |
| 83 | SDQR |  |  | + |  | + |  | + |
| 84 | SIADR |  |  | + |  | + |  | + |
| 85 | SIAVR |  |  | + |  | + |  | + |
| 86 | SIBDR |  |  | + |  | + |  | + |
| 87 | SIBVR |  |  | + |  | + |  | + |
| 88 | SMBDR |  |  |  | + | + |  | + |
| 89 | SMBVR |  |  |  | + | + |  | + |
| 90 | SMDDR |  |  |  | + | + |  | + |
| 91 | SMDVR |  |  |  | + | + |  | + |
| 92 | URADR |  |  |  | + | + |  |  |
| 93 | URAVR |  |  |  | + | + |  |  |
| 94 | URBR |  |  |  |  |  |  |  |
| 95 | URXR |  |  |  |  | + |  | + |
| 96 | URYDR |  |  |  |  |  |  |  |
| 97 | URYVR |  |  |  |  |  |  |  |
| 98 | VB1 |  |  | + | + |  |  |  |
|  | Total | 16 | 17 | 14 | 15 | 44 | 9 | 53 |

1. Includes neurons descried as sensory and amphid neurons according to WormBase.
2. Includes neurons descried as amphid neurons and amphid interneurons according to WormBase.
3. Includes all neurons descried as cord neurons (among other descriptions as well) according to WormBase.
4. Includes all neurons descried as ring motor neurons and ventral cord motor neurons according to WormBase.
5. Includes all neurons descried as ring motor neurons, ring neurons and ring interneurons according to WormBase.
6. Includes neurons descried as inner labial and outer labial neurons according to WormBase.
7. Includes all types of interneurons according to WormBase.
